# Small amounts of misassembly can have disproportionate effects on pangenome-based metagenomic analyses

**DOI:** 10.1128/msphere.00857-24

**Published:** 2025-04-29

**Authors:** Stephanie N. Majernik, Larry Beaver, Patrick H. Bradley

**Affiliations:** 1 Department of Microbiology, The Ohio State University 215854 , Columbus, Ohio, USA; 2 Infectious Diseases Institute, The Ohio State University 2647, Columbus, Ohio, USA; 3 Center of Microbiome Science, The Ohio State University 2647, Columbus, Ohio, USA; University of California Davis, Davis, California, USA

**Keywords:** metagenomics, pangenome, contamination, cirrhosis, flagella, *Lachnospiraceae*, gut microbiome, genomics

## Abstract

Individual genes from microbiomes can drive host-level phenotypes. To help identify such candidate genes, several recent tools estimate microbial gene copy numbers directly from metagenomes. These tools rely on alignments to pangenomes, which, in turn, are derived from the set of all individual genomes from one species. While large-scale metagenomic assembly efforts have made pangenome estimates more complete, mixed communities can also introduce contamination into assemblies, and it is unknown how robust pangenome-based metagenomic analyses are to these errors. To gain insight into this problem, we re-analyzed a case-control study of the gut microbiome in cirrhosis, focusing on commensal Clostridia previously implicated in this disease. We tested for differentially prevalent genes in the *Lachnospiraceae* and then investigated which were likely to be contaminants using sequence similarity searches. Out of 86 differentially prevalent genes, we found that 33 (38%) were probably contaminants originating in taxa such as *Veillonella* and *Haemophilus*, unrelated genera that were independently correlated with disease status. Our results demonstrate that even small amounts of contamination in metagenome assemblies, below typical quality thresholds, can threaten to overwhelm gene-level metagenomic analyses. However, we also show that such contaminants can be accurately identified using a method based on gene-to-species correlation. After removing these contaminants, we observe that several flagellar motility gene clusters in the *Lachnospira eligens* pangenome are associated with cirrhosis status. We have integrated our analyses into an analysis and visualization pipeline, PanSweep, that can automatically identify cases where pangenome contamination may bias the results of gene-resolved analyses.

**IMPORTANCE:**

Metagenome-assembled genomes, or MAGs, can be constructed without pure cultures of microbes. Large-scale efforts to build MAGs have yielded more complete pangenomes (i.e., sets of all genes found in one species). Pangenomes allow us to measure strain variation in gene content, which can strongly affect phenotype. However, because MAGs come from mixed communities, they can contaminate pangenomes with unrelated DNA; how much this impacts downstream analyses has not been studied. Using a metagenomic study of gut microbes in cirrhosis as our test case, we investigate how contamination affects analyses of microbial gene content. Surprisingly, even small, typical amounts of MAG contamination (<5%) result in disproportionately high levels of false positive associations (38%). Fortunately, we show that most contaminants can be automatically flagged and provide a simple method for doing so. Furthermore, applying this method reveals a new association between cirrhosis and gut microbial motility.

## INTRODUCTION

The gain or loss of individual genetic elements can drastically change microbial phenotypes, such as the pathogenicity island *cag* in *Helicobacter pylori* (1)*,* the phage-borne virulence factor *stx* in *Shigella dysenteriae* and *Escherichia coli* (2)*,* or the transposon-borne *Bacteroides fragilis* toxin BFT (3). The same applies to commensals: for example, specific strains of commensal *Bacteroides fragilis* encode genes for a capsular polysaccharide, PSA, which, in turn, evokes an anti-inflammatory response (4). This phenomenon has motivated the development of tools to measure strain-level variation in gene content. Several recent tools can estimate gene content directly from microbial communities, including MIDAS (5, 6), PanPhlAN (7, 8), and StrainPanDA (9). These methods involve aligning reads to species-specific catalogs of genes, i.e., pangenomes, and are, therefore, faster and require less coverage than metagenomic assembly.

A major drawback, however, is that these tools require many high-quality genomes to estimate pangenomes. While we have thousands of isolate genomes for some species (like *E. coli*), others may have few to none. One solution to this problem is to integrate metagenome-assembled genomes, or MAGs, which do not require pure culture and can be obtained at scale from existing data. Indeed, the construction of large MAG collections for human (10), mouse (11), and environmental (12) microbiomes has enabled much more complete pangenome estimates for a wide range of microbes, including those with few or no cultured representatives. However, genomes may also erroneously include sequences from unrelated taxa (contamination), which can then be propagated to pangenomes. Even isolate genomes are not immune to contamination (13), but because they are made by assembling and binning shotgun reads from mixed communities, MAGs are typically more likely to be contaminated by contigs from an unrelated species (14).

**Fig 1:**
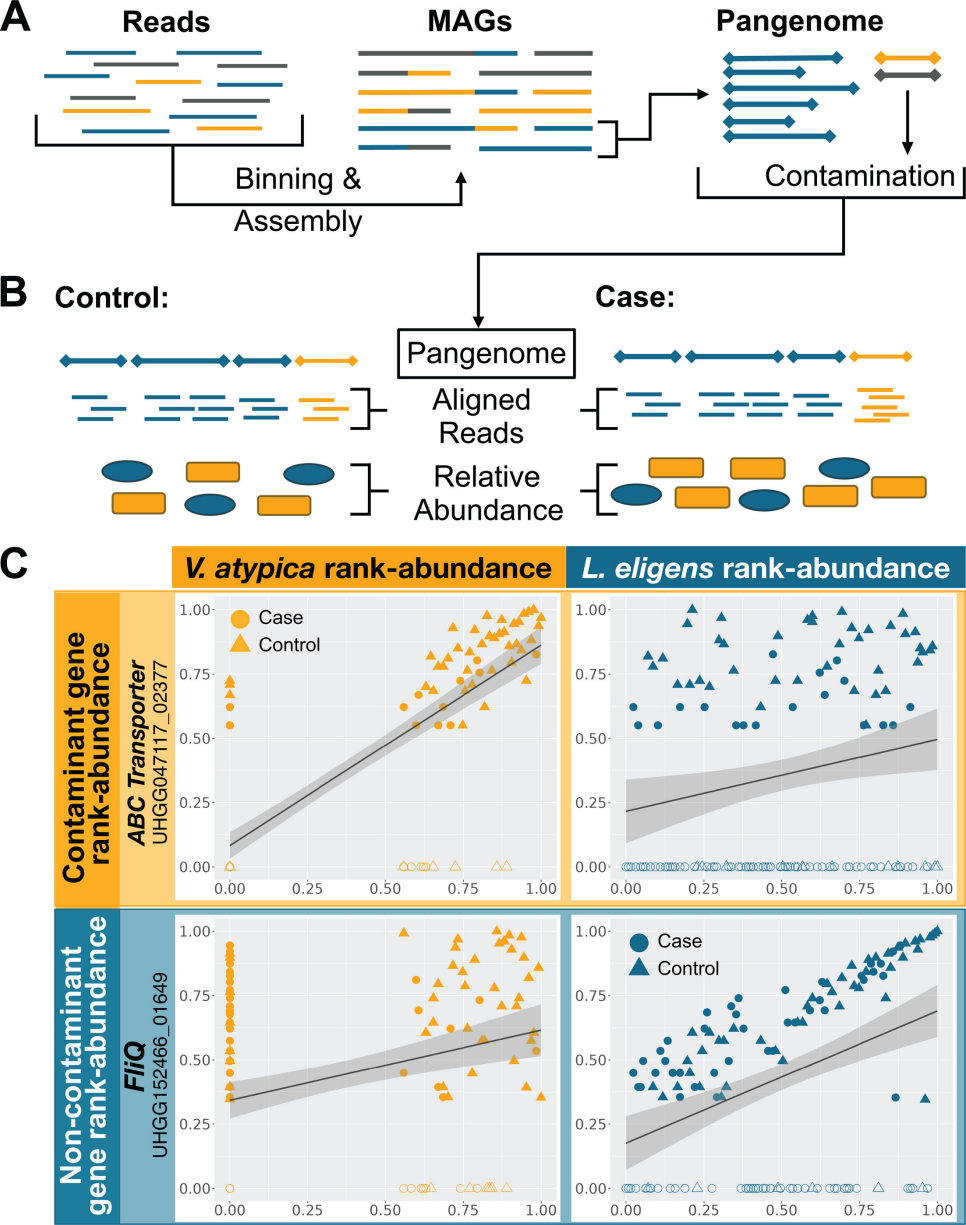
(**A**) Diagram explaining the assembly of shotgun metagenomic reads into metagenomic assembled genomes (MAGs). The true origin of each piece of DNA is represented by color. Here, contamination from the yellow species is introduced into the pangenome of the blue species. (**B**) Illustration of aligning metagenomic reads from controls (left) or cases (right) to a blue species pangenome (top line) with a small amount of contamination (yellow). The relative abundances of the blue and yellow species are visually represented below. (**C**) Example of gene-to-species correlations from the cirrhosis data set. Ranked abundances of two individual genes in the *L. eligens* pangenome (*y*-axes; rows) are plotted against ranked abundances of either *L. eligens* or a different species, *V. atypica* (*x*-axes; teal and yellow columns, respectively). Each point is one sample; samples where the gene was not detected are marked in hollow points. Using BLAST, the top gene (ABC transporter, yellow row) was found to be a contaminant in the *L. eligens* pangenome, likely from *V. atypica,* while the bottom gene (FliQ, teal row) was confirmed as a non-contaminant. Lines show best linear fit.

Tools such as CheckM (15) and GUNC (16) can be used to identify heavily contaminated MAGs and to exclude them from pangenomes. However, a typical threshold for a “high-quality draft” MAG might still allow up to 5% contamination (17). Additionally, current tools may fail to detect contaminant contigs when they do not include any highly conserved marker genes (14). Thus, a small number of contaminant genes may be introduced into the pangenome catalog (Fig. 1A). This problem has been previously identified (14) but has additional potential consequences in the context of gene-level metagenomic analyses. When reads are aligned to these contaminating genes, they will be attributed to the wrong species. Furthermore, if the source of the contamination is itself changing in abundance in cases vs controls, the contaminating gene will have the same pattern and would, therefore, be falsely identified as significantly different (Fig. 1B).

It is unknown how tolerant a pangenome-based analysis would be to these false positives. Here, we present a case study of commensal gut Clostridia in cirrhosis cases vs controls, in which we attempt to detect and correct for pangenome contamination.

## RESULTS

### Case study: commensal Clostridia gene content in cirrhosis

Numerous studies have found that gut microbial communities are altered in cirrhosis (18–21). Patients with cirrhosis show consistently lower levels of commensal Clostridia, especially those in the *Lachnospiraceae* family (18, 19), and the degree of *Lachnospiraceae* depletion correlates with severity (19). Notably, the liver and gut microbiome have a close anatomical link, exchanging metabolites via the bile ducts and portal vein. *Lachnospiraceae* produce metabolites such as butyrate that are associated with liver health, as well as with increased barrier function (22), which is diminished in cirrhosis. Finally, experimental mouse models of liver damage also indicate that *Lachnospiraceae* have beneficial effects. An oral gavage with fecal material, which enriched for *Lachnospiraceae* and butyrate in the gut, was able to reduce liver injury (23); in a mouse model of primary sclerosing cholangitis, a disease involving liver damage that can proceed to cirrhosis, reintroduction of *Lachnospiraceae* was able to reduce liver inflammation and fibrotic lesions (24). We were, therefore, interested in identifying genetic elements that could allow *Lachnospiraceae* to persist in the cirrhotic gut, as this could help drive the selection of novel probiotics.

To identify such genes and genetic elements, we re-analyzed short-read shotgun metagenomes from a large case-control study of liver cirrhosis from multiple causes (25) (Qin et al.; *n* = 123 cases and *n* = 114 controls). We used MIDAS2 (6) to assign reads to the pangenomes of species derived from the Universal Human Gut Genome database, or UHGG (10), which includes both isolate sequences and metagenomic assemblies. Next, we applied Fisher’s exact test to identify genes from *Lachnospiraceae* that were differentially prevalent in case vs control metagenomes, using the DiscreteFDR method to adjust for multiple comparisons (26). Setting a false discovery rate (FDR) cutoff of 5% yielded 86 significant genes in six *Lachnospiraceae* species.

To find potential homologs of these genes, we performed a BLAST search of their translated sequences against NCBI’s “nr” database (27). We were surprised to find that in 33 out of 86 cases, the closest matches were not found in *Lachnospiraceae* or even other Clostridia. Rather, they were found in distantly related taxa such as *Veillonella* (class Negativicutes), *Haemophilus* (class Gammaproteobacteria), and *Streptococcus* (class Bacilli). To confirm these taxonomic distributions more rigorously, we also matched our sequences to fine-grained groups of orthologs from the EggNOG database (28). This was possible because gene sequences from UHGG had already been clustered into protein families at different amino acid identity thresholds in the companion UHGP database (10); these, in turn, were annotated using several methods including the tool eggNOG-mapper (29). Our results largely matched what we observed using BLAST (Table 1; Table S1).

**TABLE 1:** PanSweep results for all *Lachnospiraceae* species that had genes significantly associated with cirrhosis^*a*^

MIDAS gene ID	Isol	MAG	Predicted taxonomic group	Pred. protein name	FDR	Pangenome species	Species with max correlation value	Rank (1st sp. in fam)	Sp. Corr. rank	BLAST Contam	Notes
UHGG064838_00419	27	2064			*1.9E−3*	Anaerostipes hadrus	Veillonella atypica	12	12	No	
UHGG016431_01839	0	3	Eubacteriaceae	cheV	1.1E−2	Lachnospira rogosae	Lachnospira sp900316325	1	536	No	
UHGG063379_01966	4	1,413	unclassified Clostridiales	epsG	1.1E−2		Lachnospira sp900316325	1	426	No	
UHGG064037_00369	0	1	Eubacteriaceae	glmS	1.1E-2		Lachnospira sp900316325	1	1343	No	
UHGG108644_01598	0	9	Clostridia	ybbR	1.1E−2		Lachnospira sp900316325	1	635	No	
UHGG109369_01803	0	1	Eubacteriaceae	IV02_08645	4.5E−2		Lachnospira sp900316325	1	757	No	
UHGG151972_01002	0	1	Clostridiaceae	els	1.1E−2		Lachnospira sp900316325	1	570	No	
UHGG154988_02076	0	1	Eubacteriaceae		1.1E−2		Lachnospira sp900316325	1	655	No	
UHGG188302_00118	0	1	Eubacteriaceae	yabB	1.1E−2		Lachnospira sp900316325	1	453	No	
UHGG195023_01285	0	1	Ruminococcaceae	arsA	1.1E−2		Lachnospira sp900316325	1	778	No	
UHGG000117_00039	15	979	Clostridia		1.9E−2	Roseburia inulinivorans	Roseburia inulinivorans	1	1	No	
UHGG000117_02863	14	839	Clostridia		3.7E−2		Roseburia inulinivorans	1	1	No	
UHGG000117_03451	6	101			1.2E−2		Prevotella buccae	6	887	No	
UHGG000355_03097	4	71	Clostridia		4.0E−2		CAG-882 sp003486385	1	92	No	
UHGG001770_01542	1	115	Paenibacillaceae		3.7E−2		Haemophilus_D	9	240	No	
UHGG004804_02592	11	813	Clostridia		4.0E−2		Staphylococcus xylosus_B	5	193	No	
UHGG006614_00504	2	46			4.5E−2		Granulicatella sp001071995	2	4	No	Some fragmentation
UHGG027032_02679	0	1	Eubacteriaceae		3.7E−2		Streptococcus sp000411475	2	696	No	
UHGG032492_02428	0	1	Pasteurellales	CP_0066	5.8E−3		Haemophilus_D parainfluenzae	85	116	Yes	
UHGG083479_02753	0	1	Pasteurellales	yfhL	1.5E−2		Haemophilus_D parainfluenzae	74	106	Yes	
UHGG115855_01382	0	3			4.0E−2		Roseburia inulinivorans	1	1	No	
UHGG125279_02764	8	257			4.0E−2		Prevotella	6	48	No	Some fragmentation
UHGG137889_03520	0	1	Pasteurellales	aroE	3.7E−2		Haemophilus_D parainfluenzae	82	93	Yes	
UHGG155662_03633	0	1	Negativicutes		5.8E−3		Haemophilus_D	56	86	Yes	
UHGG155662_03708	0	1	Negativicutes	mtnU	4.5E−2		Veillonella parvula	91	467	Yes	
UHGG155662_03778	0	1	Negativicutes		5.8E−3		Haemophilus_D	31	92	Yes	
UHGG155827_01231	0	1			1.5E−2		Veillonella atypica	21	100	Yes	
UHGG198550_01569	15	838	Clostridia	nifJ	3.7E−2		Veillonella atypica	67	123	No	
UHGG200056_01832	0	1	Negativicutes	prs	3.3E−5		Veillonella atypica	74	109	Yes	
UHGG200056_01833	0	1	Negativicutes	glmU	2.0E−3		Veillonella atypica	86	538	Yes	
UHGG200056_01834	0	1	Negativicutes	purR	2.0E−3		Veillonella	79	296	Yes	
UHGG200056_01835	0	1	Negativicutes		3.7E−5		Veillonella atypica	75	255	Yes	
UHGG000216_02074	16	75	Clostridia		3.1E−3	Lachnospira eligens_B	Lachnospira eligens_B	1	1	No	
UHGG000216_02420	20	2811			3.8E−2		Lachnospira eligens_B	1	1	No	
UHGG001288_02675	21	2,809	Eubacteriaceae	pip	2.2E−3		Streptococcus sp001556435	2	2	No	
UHGG011927_00007	0	8			1.3E−2		CAG-882 sp003486385	1	239	No	
UHGG029873_02068	0	1			8.8E−4		Streptococcus parasanguinis	81	224	Yes	
UHGG030659_00967	21	2,984	Eubacteriaceae	prfB	1.4E−2		Veillonella atypica	65	262	Yes	Chimeric
UHGG032185_00468	21	2,930	Eubacteriaceae	atpA	1.6E−2		Veillonella infantium	71	71	Yes	Chimeric
UHGG033855_01831	0	7			4.4E−2		Lachnospira sp900316325	1	1358	No	
UHGG033855_01832	0	4	Eubacteriaceae	tkt	4.7E−2		Lachnospira sp900316325	1	804	No	
UHGG036212_01418	21	2,981			3.2E−2		Lachnospira sp900316325	1	1498	No	
UHGG038364_02001	0	3	Desulfovibrionales	pqqL	4.4E−2		Bilophila wadsworthia	11	1435	Yes	
UHGG041746_02388	21	2,403	Eubacteriaceae		7.6E−3		Veillonella	96	169	Yes	Chimeric
UHGG041746_02389	0	1	Bacteria		1.1E−5		Veillonella infantium	77	218	Yes	
UHGG041746_02390	0	1			3.9E−6		Veillonella atypica	66	93	Yes	
UHGG047117_02375	6	243			1.3E−2		Veillonella atypica	48	122	Yes	
UHGG047117_02376	0	1	Negativicutes	rpsO	4.9E−6		Veillonella atypica	81	102	Yes	
UHGG047117_02377	0	1	Negativicutes		3.2E−5		Veillonella atypica	81	149	Yes	
UHGG047117_02378	0	1	Negativicutes		1.8E−5		Veillonella atypica	84	202	Yes	
UHGG047117_02379	0	1	Negativicutes	XK27_00670	3.9E−6		Veillonella infantium	81	188	Yes	
UHGG047117_02380	0	1	Negativicutes	XK27_00670	5.2E−4		Veillonella	78	181	Yes	
UHGG047117_02381	0	1			8.0E−4		Veillonella infantium	81	109	Yes	
UHGG047117_02382	0	1	Negativicutes	apeA	5.0E−6		Veillonella infantium	76	143	Yes	
UHGG047117_02383	0	1	Negativicutes		5.5E−3		Veillonella infantium	81	204	Yes	
UHGG051001_01727	0	7			4.7E−2		Lachnospira sp900316325	1	1515	No	
UHGG052248_01872	0	1			2.1E−2		ER4 sp000765235	6	710	Yes	
UHGG055467_01854	21	2,893	Negativicutes	dapL	1.1E−4		Veillonella atypica	79	248	Yes	
UHGG057388_00211	21	1,810			3.2E−2		CAG-882 sp003486385	1	117	No	
UHGG063307_00097	0	2	Eubacteriaceae	ftsZ	5.5E−3		Lachnospira sp900316325	1	961	No	
UHGG083468_01778	0	17			1.4E−2		Lachnospira eligens_B	1	1	No	Poor-quality alignments
UHGG108323_00300	0	3	Clostridia		4.7E−2		Lachnospira sp900316325	1	839	No	Also aligns to Inovirus
UHGG108348_02595	0	22	Clostridia		1.4E−2		CAG-882 sp003486385	1	70	No	
UHGG148769_00831	21	2,896	Eubacteriaceae	yloV	4.3E−2		Lachnospira eligens_B	1	1	No	
UHGG148794_01632	0	3	Eubacteriaceae	fliW	5.5E−3		Lachnospira sp900316325	1	1258	No	
UHGG152430_02393	21	2,240	Blautia	rpsM	3.6E−2		CAG-882 sp003486385	1	9	No	
UHGG152466_01649	19	2,568	Eubacteriaceae	fliQ	3.4E−2		Lachnospira eligens_B	1	1	No	
UHGG181581_00481	0	1	Pasteurellales	trmJ	8.8E−3		Haemophilus_D parainfluenzae	61	288	Yes	
UHGG192308_01194	21	2,862	Eubacteriaceae		4.9E−3		Veillonella infantium	51	54	Yes	Chimeric
UHGG210755_00357	0	39	Clostridia		3.7E−2		Lachnospira sp003537285	1	2	No	
UHGG210793_02030	5	311			5.5E−5		Veillonella parvula	12	41	No	
UHGG210928_01151	0	1			2.1E−5		Streptococcus sp001556435	79	108	Yes	
UHGG228006_01743	0	3	Eubacteriaceae		8.8E−3		Lachnospira sp900316325	1	980	No	
UHGG230652_00809	0	1			1.4E−2		Faecalicatena	1	561	No	
UHGG235946_01311	21	2,799			3.8E−3		Lachnospira eligens_B	1	1	No	
UHGG239171_01612	0	1	unclassified Lachnospiraceae		5.5E−3		CAG-882 sp003486385	1	18	No	
UHGG249173_01879	15	921	Butyrivibrio	arsC1	3.2E−2		Absiella sp000165065	2	5	No	
UHGG258864_02598	21	2,809			3.3E−2		Lachnospira sp900316325	1	1484	No	
UHGG031386_02238	0	1	Erysipelotrichia		5.0E−2	Acetatifactor sp900066565	UBA9502	1	741	Yes	
UHGG046826_00950	0	1	Oribacterium	argE	1.2E−2		Oscillibacter	17	204	No	
UHGG047729_02814	0	1	Oscillospiraceae		5.0E−2		CAG-170 sp000432135	26	1539	No	
UHGG000135_01093	26	1,018	Blautia	ftsH	3.1E−2	Faecalicatena gnavus	Faecalicatena gnavus	1	1	No	
UHGG005482_00240	0	1	Blautia	pcs	4.1E−2		CAG-103 sp000432375	3	1079	No	
UHGG076937_00800	0	1	Blautia	ispG	4.1E−2		Blautia_A massiliensis	1	1095	No	
UHGG169515_01624	0	2	Blautia	nhaC	4.1E−2		CAG-103 sp000432375	2	1029	No	
UHGG187977_00187	0	1	Clostridia		4.1E−2		Blautia_A massiliensis	1	981	No	

^*a*^ Columns include (1) the ID of the gene in the MIDAS2 UHGG database (available through MIDAS2, https://midas2.readthedocs.io/); (2–3) the number of isolate and MAG genomes in UHGG that contain the gene; (4–5) the predicted taxonomic range and predicted name of the corresponding UHGP-90 protein cluster (available at https://ftp.ebi.ac.uk/pub/databases/metagenomics/mgnify_genomes/human-gut/v1.0/uhgp_catalogue/), using UHGG’s EggNOG-mapper annotations; (6) the false discovery rate *q*-value of the gene; (7) name of the pangenome species in the MIDAS2 UHGG database (shown once per set of genes from the same pangenome); (8–9) whether the species with highest rank correlation to the gene matched the lineage of the pangenome species to at least the family level, and what the highest ranked species was; (10) the highest rank of species in the same family of the originating pangenome; (11–12) whether the BLAST results indicated contamination or not, and any other observations from the BLAST results (e.g., if the gene appeared to be chimeric, or if the alignments were generally poor quality).

Qin et al. (25) as well as other studies (18, 21) found that the oral taxa *Streptococcus, Haemophilus,* and especially *Veillonella* were more abundant in cirrhotic gut microbiomes. Thus, the apparent increase in these genes’ copy number could be an artifact caused by increased *Veillonella, Haemophilus,* or *Streptococcus* relative abundance. Indeed, the ABC transporter gene UHGG047117_02377, an example of a likely contaminant, correlates better with the abundance of *Veillonella atypica* than the species of the pangenome where it appears, *Lachnospira eligens*. The opposite trend can be seen for the flagellar gene FliQ (UHGG152466_01649) in *L. eligens* (Fig. 1C).

Out of the 33 putative contaminant genes, 26 were present in only a single *Lachnospiraceae* assembly. According to CheckM, these 13 single assemblies had a mean contamination estimate of 0.22% (interquartile range: 0%–0.24%). This is actually below the average contamination estimate of all *Lachnospiraceae* genomes (1.4%; IQR: 0.19%–2.2%). Overall, assemblies containing contaminant genes had somewhat higher estimated contamination than other assemblies from the same species, but all were still below the 5% threshold that CheckM recommends (15) (Fig. S2). A recent revision of UHGG (30) uses an additional, more sensitive chimera detection tool called GUNC (16), but this did not flag any of the assemblies bearing the contaminant genes. These results demonstrate that in pangenome-based analyses, even unremarkable amounts of assembly contamination can lead to pathological results when the contamination source is itself correlated with the phenotype of interest. Furthermore, because misassembly is most likely to result from microbes found in the same environment, this scenario may be more common than it first appears.

### An analysis and visualization tool to identify potential contaminants

To help researchers identify potential contaminants that may obscure pangenome-based analyses, we created an workflow called PanSweep, available as an R package, that integrates the tests we performed above and visualizes the results. This workflow helps flag contaminants in three ways: (i) loading and comparing gene annotations transferred from the EggNOG database (28, 29); (ii) correlating gene and species read counts; and (iii) visually representing patterns of gene co-occurrence across metagenomic samples. While PanSweep was designed to analyze the results from the tool MIDAS2 (6), the contamination test based on gene-species correlation can be invoked separately from the rest of the pipeline and only requires the user to provide abundance matrices and the taxonomic annotations of each species.

First, each gene is associated with its UHGP-90 cluster (i.e., protein sequences clustered at 90% amino acid identity). EggNOG-mapper (29) was previously used to link UHGP-90 centroid sequences to the most taxonomically specific EggNOG orthologous group (OG) in EggNOG v5.0 (see Materials and Methods). We report the taxonomic label of this OG (e.g., *Clostridiales*) so that the user can assess whether the lineages match. Second, per-gene read counts are correlated with all species abundances using Spearman’s *ρ*. For each gene, the correlations to each species are then ranked from the highest to lowest. We report the rank of the most-correlated species that matches the gene’s expected lineage (based on the pangenome in which it is found). We have also implemented a “conditional” version of this test, in which only samples where the gene is present are used to compute the correlation (see Materials and Methods). Finally, to determine which genes may be part of the same contig or mobile element, we calculate a similarity matrix of gene co-occurrence using the Jaccard index. This Jaccard matrix is visually represented as a clustered heatmap and as ordination plots: the user can choose between UMAP (31), NMDS (32, 33), and PCoA.

The gene-to-species correlation test was especially consistent with our BLAST analysis. Out of the 39 genes where the most correlated species was in the same family, only one showed evidence of contamination via BLAST, a false positive rate of 2.6%. This only increased to 2/50, or 4%, when we allowed a family lineage match anywhere in the top 10 most-correlated species. Conversely, in the 28 cases where a lineage match did not occur in the top 50 most-correlated species, 27 were confirmed to be contaminants by BLAST (a true positive rate of 96%). We can summarize the performance across all cutoffs by calculating the area under the receiver-operator characteristic curve (AUROC), where 1 indicates perfect prediction and 0.5 is random. The correlation lineage test had an AUROC of 0.97, indicating near-perfect classification (Fig. S2). In contrast, a variation where we only considered exact species matches was not effective (AUROC = 0.53, near random). Using the conditional correlation test, performance was nearly the same for family-level matches (AUROC = 0.97) but improved markedly at the species-level (AUROC = 0.75).

EggNOG taxonomic annotations were also highly predictive of contamination (AUROC = 0.86). All 19 cases in which EggNOG predicted a protein’s range to be in the Negativicutes or Pasteurellales were confirmed by BLAST analysis to be contaminants, with similar sequences mostly or exclusively outside of the *Lachnospiraceae*. EggNOG annotations to *Eubacteriaceae*, Clostridia (and similar taxa), *Lachnospiraceae*, or *Ruminococcaceae* tended to be legitimate: 28 of these were likely true *Lachnospiraceae* genes, while 4 appeared to be contaminants. However, EggNOG annotations were not available or very general (Bacteria) for 24 cases, 8 of which were likely contaminants based on BLAST.

**Fig 2:**
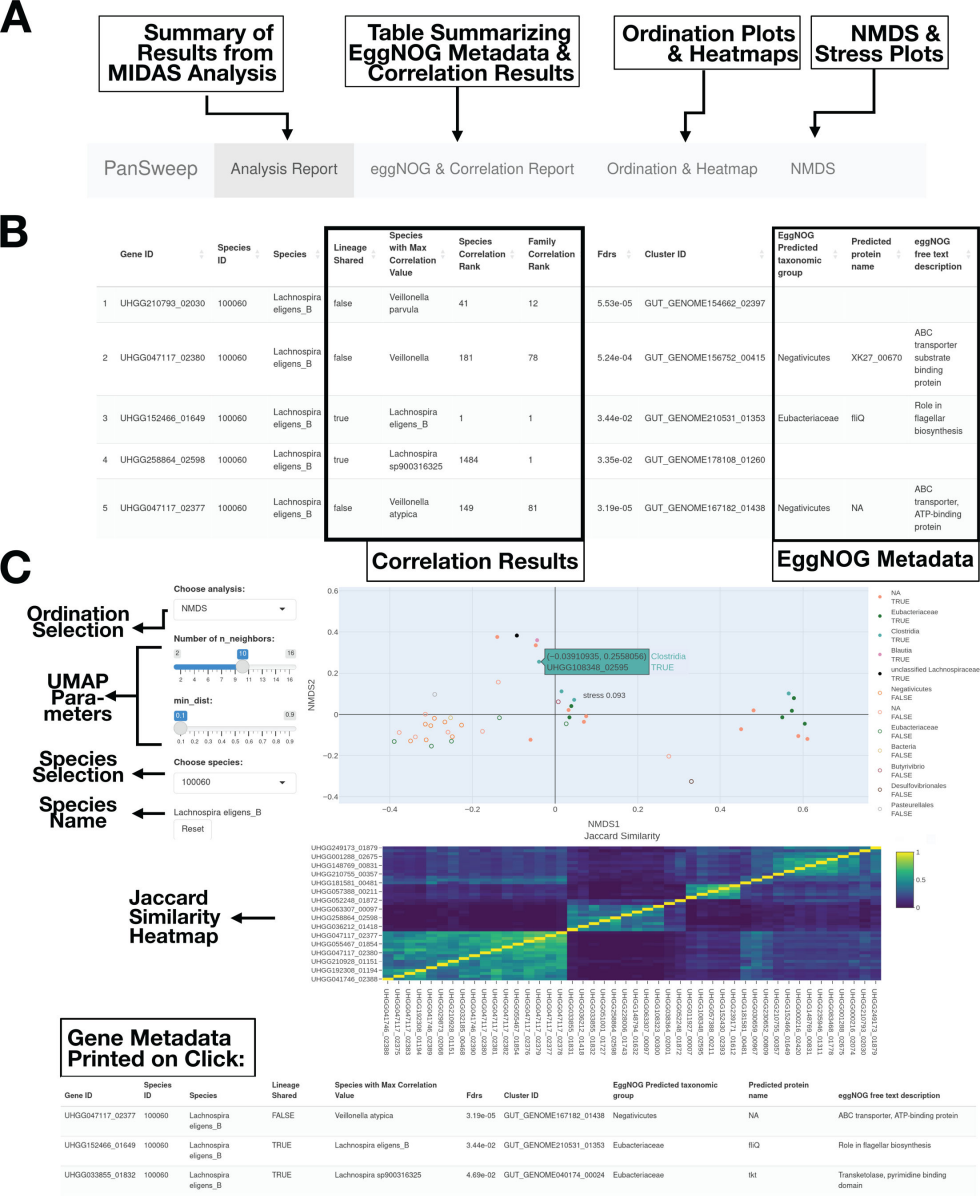
(**A**) Annotated screenshot explaining the four main tabs of PanSweep. (**B**) Annotated screenshot showing the first five rows of a table of significant genes. The columns from left to right show the gene ID in the UHGG database, the species ID from MIDAS, the species name, whether the most correlated species shared a family with the pangenome species, which species was most correlated to the gene, the calculated FDR, the UHGP-90 cluster identity, the predicted taxonomic group from the EggNOG database, and the predicted protein name from EggNOG. (**C**) Ordination plot and heatmap for *L. eligens*. In this static visualization, the interactive NMDS plot of gene presence-absence (Jaccard distance) shows the label of a gene that would be displayed on mouse-over. The sliders allow users to modify the UMAP parameters. The clustered Jaccard similarity matrix of gene presence-absence profiles across samples is shown as a heatmap below the NMDS. Finally, gene metadata for any selected genes is printed at the bottom.

Gene-to-gene correlation can also help identify contaminants. When we calculated Jaccard dissimilarity between all the significant *Lachnospira eligens* genes and examined the co-occurrence heatmap, 13 of the putative contaminants formed a single well-defined cluster. Nine of these were, in fact, adjacent features on a single contig in the same MAG, GUT_GENOME047117 (Fig. 2C). In contrast, 25 non-contaminant genes in *L. eligens* were not correlated with the genes that failed, and not all were correlated with one another (Fig. 2C).

Overall, 38% (33/86) of our significant results were likely to be the result of contamination. Importantly, statistical significance did not predict contamination and, in fact, was anti-correlated (AUROC = 0.21), meaning that stricter cutoffs would actually make the problem worse. Considering just those genes with adjusted *P*-values of below 0.01, 72% were contaminants (23/32).

### Removing contaminants enhances biological signal

The majority of the 25 *L*. *eligens* non-contaminant genes had no predicted protein name or description from EggNOG, highlighting the need for improved functional annotation of gut microbial genomes (10). Strikingly, however, out of the nine genes that had any EggNOG free-text annotation, two were directly involved in chemotaxis, and two more had a plausible role in this process. A gene annotated as FliQ, part of the type III secretion system that helps to assemble the flagellar hook-basal body (34), was found in 80% of cases (41/51) but only 48% of controls (37/77). In contrast, a gene annotated as the flagellar regulator FliW had the opposite trend, being found in 26% of controls (20/77) and zero cases (0/51). An unnamed third gene, UHGG228006_01743, had a similar pattern to FliW (19/77 controls, 0/51 cases). Searching this third gene’s amino acid sequence in InterPro (35) revealed the presence of a GGDEF diguanylate cyclase domain (36) and a PAS sensory domain (37). (While the EggNOG annotation also suggested the presence of an EAL domain, we did not find any evidence for this in InterPro.) This combination of domains suggests the possibility that in response to the presence or absence of an intracellular signal, the protein UHGG228006_01743 may synthesize cyclic di-GMP, a metabolite that typically regulates the transition between motility and other behaviors like biofilm formation (38). Another unnamed diguanylate cyclase was also differentially prevalent but was more frequently seen in cases (1/77 controls vs 13/51 cases).

To investigate why we saw both positive and negative associations with cirrhosis, we plotted the estimated copy numbers of all genes in the *L. eligens* pangenome described in EggNOG annotations as flagellar, plus the two diguanylate cyclases named above (Fig. S3). This analysis revealed large, distinct clusters of correlated flagellar genes, which we expected since many of these genes are located in the same genomic neighborhoods. One of these clusters included FliQ as well as nine other genes and had copy numbers that were higher in cirrhosis (adjusted *P* = 0.027, see Materials and Methods). A separate cluster included the FliW we identified as significant, as well as the unnamed GGDEF-PAS gene and three other genes, and was strongly enriched in controls (adjusted *P* = 1.1 × 10^−6^, see Materials and Methods). This analysis shows that strain variation in *L. eligens* flagellar loci can differentiate cirrhosis cases and controls. Furthermore, it illustrates that by eliminating likely contaminants, functional linkages—in this case, between *Lachnospiraceae* motility and the cirrhotic gut—can become clearer and more consistent.

## DISCUSSION

This study illustrates an issue with pangenome-based analyses of metagenomes: even small amounts of pangenome contamination can cause the results to be overwhelmed by contaminants. The above case study demonstrates that this problem is not merely theoretical but occurs even in non-pathological, real-world data sets. This problem is also distinct from another important, previously raised issue called cross-mapping, in which reads do not map uniquely to a single gene (39). In our case, it seems the contaminant genes are being measured accurately, as they correlate best to their likely source. The problem is, rather, that trace pangenome contamination leads to copy number variants being attributed to the wrong species. That said, the correlation-based approach we describe may also help to flag cases for which pervasive cross-mapping from a gene in a distantly related species has made quantification unreliable.

In this study, the most common sources of contaminant genes were from the *Veillonella* and *Staphylococcus* genera. *Staphylococcus* and *Veillonella* increase in relative abundance in cirrhosis cases, and in fact, increased oral taxa correlate with disease and dysbiosis more broadly (40). A recent explanation for this phenomenon is that oral taxa are actually a biomarker of lower total gut biomass, as they will appear to increase in relative abundance when total gut biomass drops (41). Contamination from these species may, therefore, be especially problematic for studies of other diseases and the gut microbiome. Strikingly, *Veillonella* was also the source of contamination in a case study (14) used to illustrate that the marker-gene approach used in CheckM (15) failed to identify significant chimerism in an oral *Saccharibacteria* MAG.

The contamination we observed originated mainly from single MAGs, suggesting that dropping infrequently observed gene clusters from the pangenome, an alternative strategy that can be used in the forthcoming MIDAS3 (42), could also be a helpful approach. However, while thousands of MAGs exist for some genera, only a handful may exist for others. Furthermore, in the species *Lachnospira rogosae,* six out of nine *non*-contaminant genes were only observed in a single MAG; dropping these would, therefore, decrease sensitivity. StrainPGC, a strain deconvolution tool that uses the output of MIDAS3 (42), also uses correlation to refine its estimates of strain genome content. Furthermore, incorporating cross-sample correlation into the process of binning itself has been shown to effectively identify and screen out contaminants (43). The current study reinforces this strategy, and shows it may be productive to apply a similar technique at the species as well as the strain or genome level.

We have not yet comprehensively tested the correlation test’s performance on other taxa or data sets. That said, as a quick test of its generalizability, we did re-run PanSweep on the same data set for the species *Bacteroides thetaiotaomicron*, a Gram-negative commensal in a different phylum. We then performed BLAST analysis on a random set of 30 significant genes. Similar to the *Lachnospiraceae*, we obtained a high AUROC (0.91) and found that no genes passing the correlation lineage test were contaminants (0/15, 0% FPR), while all genes with a maximum family rank of at least 50 were (7/7, 0% FNR). This suggests PanSweep can be productively applied outside the *Lachnospiraceae*.

One factor that could potentially influence the results of the correlation test is population structure, as co-existing subpopulations could make the total species abundance less informative. The gene-to-gene similarity matrix we present could help researchers identify such cases. Also, while we did not observe a major difference in this study, the “conditional” correlation test could become more important for taxa with large accessory genomes and substantial population structure. We would, therefore, still encourage users to spot-check individual genes flagged by the tests in PanSweep.

Our results may underestimate the utility of comparisons to EggNOG taxonomic ranges. EggNOG and UHGG use different taxonomic databases: EggNOG uses NCBI Taxonomy, which emphasizes validly-published taxon names, whereas UHGG (and the MIDAS2 UHGG database) uses GTDB, which emphasizes phylogenetic coherence. It is particularly difficult to compare lineages across these two databases for *Lachnospiraceae* or other commensal *Clostridia*: many have only recently been re-classified, based on phylogenomic information, from entirely different orders, families, and genera. This historical lack of phylogenetic coherence in the *Clostridia* could also have affected how the EggNOG OGs themselves were constructed. However, official species classifications are gradually changing to reflect new phylogenetic data, and these two databases will likely continue to converge in the future. Furthermore, our results do show that even in a particularly challenging case, EggNOG annotations can still be useful for identifying contamination from distant clades. These annotations can also be used to evaluate an entire pangenome: taking *L. eligens* as an example, 22% of all accessory genes had discrepant EggNOG annotations (compared to 1.1% of core genes present in ≥95% of genomes), but this dropped to 7.6% when considering only genes found in isolate genomes (1.5% of core genes), indicating that MAGs are much more likely to contribute genes with anomalous phylogenetic distributions.

It is possible that some genes flagged as contaminants could in fact be instances of recent horizontal gene transfer (HGT) events, especially as HGT appears to be common in the gut (44). However, while we cannot rule this out, we believe that typically, HGT would be a less-likely explanation. First, we only flag cases where lineages do not match at the family level or above, and HGT is much less common across this distance than, e.g., within a genus (44). Next, to be detected in the recipient, an HGT event would have to also be captured in our pangenome database, or else any reads from the recipient would only align to the donor’s pangenome. Finally, even if a real HGT event were captured in the database, for it to affect the correlations we observe, it would have to happen independently in a significant proportion of the samples being studied. Otherwise, most reads in most samples would come from the donor species, not the recipient. One exception where we might suspect recent, repeated HGT would be in the case of genes that are highly mobile and under strong selection. Such genes would be likely to have broad taxonomic distributions (e.g., across all Bacteria, not specific to a particular family such as *Veillonellaceae*) and to be enriched for specific functions [e.g., antibiotic and metal resistance, virulence, integration, and conjugation (44)]. In the *L. eligens* pangenome, for example, genes with such a broad predicted distribution were rare (0.18%).

While our work focuses on a particular case study in the human gut microbiome, we expect that this problem will also be an issue in other environments, especially as more and more metagenomic assemblies are generated. In fact, many environments have markedly fewer available isolate genomes than the human gut and, thus, depend even more on assembly. Certain genomic features may also pose problems: for example, mobile elements like prophage, which can contain repetitive sequences and/or be present in multiple copies, are known to complicate metagenomic assembly (45).

The method we propose of using correlation together with taxonomic information appears to be effective, is faster at identifying contaminants than alignments against large databases (yet still agrees with the results), and could potentially be applied broadly across different environments and study designs. We see this as work as complementary to other recent methods that directly improve MAG quality by detecting and filtering contaminants in each genome, such as the deep-learning tool Deepurify (46) and the fast hash-based method FCS-GX (47). As demonstrated here, removing pangenome contaminants allows researchers to home in on the most promising new associations between microbial gene function and disease, a critical step in moving from species-level associations to testable hypotheses.

## MATERIALS AND METHODS

### MIDAS2 analysis

Sequencing runs associated with BioProject ID PRJEB6337 from NCBI’s Sequence Read Archive (48) were downloaded and converted to FASTQ files. We used BBDuk to trim reads from the right below a Phred score of 20, discarding reads that were trimmed below 60 bp (49). Samples with fewer than 1 × 10^6^ reads after trimming and filtering were discarded (*n* = 22), leaving 292 samples. These samples represented 237 subjects, with 123 cases and 114 controls. Trimmed FASTQ files were analyzed using MIDAS2 (6) with the UHGG database (10). Briefly, MIDAS2 was first used to compute species coverage levels across all samples. As recommended, all species with at least 2× marker coverage and 50% uniquely mapped markers in some sample were then selected for copy number variant quantification with MIDAS2. Note that because of this coverage threshold, the total number of subjects in each group differed depending on the species. Copy number estimates for all species in the *Lachnospiraceae* were retained for subsequent analysis. The steps above were performed using a Snakemake pipeline (50).

### Determination of differentially prevalent genes

To identify genes for further study, we focused on prevalence, or the rate that a gene was detected. We used presence/absence estimates generated by MIDAS2. Genes with significant differences in prevalence across samples were identified with Fisher’s exact test, and the resulting *P*-values were adjusted for multiple comparisons using the DiscreteFDR method (26). Adjusted *P*-values at or below 0.05 were counted as significant.

### BLAST analysis

Nucleotide sequences for each gene were obtained from the MIDAS2 pangenomes, which had been clustered at 95% nucleotide ID. Each sequence was first analyzed using NCBI Megablast, with default parameters, against the nt database (51). If no sequences were identified, or the results were ambiguous, the sequence was reanalyzed first using discontiguous Megablast, BLASTN, and finally using translated BLASTX against the nr database. We then used the reported taxonomy information to determine whether most of the best hits were annotated with species falling within the *Lachnospiraceae* family.

In most cases, the taxonomic distribution of the gene was unambiguous, with only a few requiring additional follow-up (see Fig. S4 and S5 for examples). We flagged genes as potentially “chimeric” if one region of the sequence aligned well to *Lachnospiraceae,* while a second region aligned well to a divergent taxon such as *Veillonella,* with only a small overlap aligning well to both. These cases suggest possible misassemblies, giving rise to chimeric genes in the pangenome. We counted these genes as contaminants; however, we note that even if they did not derive from misassembly, they should still likely be excluded from further analysis, because we would expect reads originating from both taxa to align well to the gene. This is related to the “cross-mapping” artifact previously described by Zhao et al. (39).

### PanSweep pipeline

The PanSweep pipeline is written in R (52) and consists of two main parts: a function that performs offline analysis and generates files for visualization, and a function that allows for real-time visualization of the results.

The offline analysis function has three components:

1. Identifying differentially prevalent genes;
2. Calculating Jaccard (dis)similarity of gene presence-absence profiles and performing dimension reduction; and
3. Performing the correlation lineage test.

Part 1 identifies differentially prevalent genes between cases and controls based on the output of MIDAS2 and provided sample metadata as described above. In Part 2, for species with three or more significant genes, Jaccard similarities between gene presence-absence profiles are pre-calculated for each gene pair. Then, the Jaccard similarity matrices are converted to dissimilarity matrices, and NMDS, UMAP, and PCoA are used to provide two-dimensional representations (53, 54).

In Part 3, for each significantly associated gene, the gene counts (number of reads mapping to the gene) are correlated to the relative abundance of each species using Spearman’s *ρ*. We then rank these species-to-gene correlations in descending order, such that a rank of 1 is the highest. Next, the lineage of each species is compared to the lineage of the species where the gene was annotated. We report the highest rank where the lineages match. We consider two ways of matching taxa: one where lineages are matched at the family level, and one where they are matched at the species level. Finally, in addition to the “overall” version, which considers all samples where the gene or species was measured, we have also implemented a “conditional” version of this test, which performs Spearman’s correlation over *only* the samples in which a gene has been detected. The intuition behind this version is that by definition, genes in the accessory genome of a given species may not always be present, but that when that gene *is* detected, its abundance should correlate with the species it comes from.

The real-time visualization tool is a Shiny (55) application that loads in metadata about each gene, plots the above gene-to-gene similarity matrices and ordinations, and displays summary tables showing EggNOG-derived phylogenetic range predictions as well as the results of the lineage test. Jaccard similarity matrices are first hierarchically clustered to group similar genes together (Euclidean distance, complete linkage) and then plotted interactively using plotly (56) with viridis (57) coloring. The user can select genes to highlight on this heatmap; information about selected genes will then appear below. The type of ordination (NMDS, UMAP, or PCoA) can also be set in real-time by the user. Additionally, the UMAP parameters “n_neighbors” and “min_dist” can be user-adjusted via toggle bars, with “n_neighbors” ranging from two to one-third the total number of genes (rounded up to the nearest whole number), and “min_dist” ranging from 0.1 to 0.9 at intervals of 0.1. The stress plot from the NMDS analysis is also provided.

EggNOG orthologous group (OG) predictions have been previously made for all UHGP-90 protein clusters (10) using EggNOG-mapper (29), allowing us to retrieve the predicted taxonomic range. Briefly, EggNOG first clusters sequences via an all-versus-all alignment, creating groups of proteins that are more similar to each other than to other proteins. OGs of these proteins are defined hierarchically at different nodes in the species tree, which represent speciation events: each OG contains the sequences that descend from that node, including any paralogs that formed after that speciation event (in-paralogs), but not paralogs that formed before (out-paralogs) (58). EggNOG-mapper finds the best hit of a query sequence in EggNOG and then returns the most taxonomically-specific OG that contains this hit (29).

To allow the application to efficiently find which UHGP-90 cluster a given gene corresponded to, the tab-separated mapping file provided in UHGP was converted into a Parquet database, with gene IDs stored as integers. We similarly constructed a Parquet database from the eggnog-mapper output provided for UHGP-90 protein clusters. These files are provided with the application. The R package “arrow” (59) is used to read and write Parquet-format files.

### Evaluation

EggNOG taxonomic ranges were calculated using the NCBI taxonomy database and, therefore, could not be directly compared to species annotations from UHGG, which uses the GTDB database. We considered annotations to taxa that did not include any *Lachnospiraceae* (*Paenibacillaceae*, Pasteurellales, Negativicutes, Desulfovibrionales, *Erysipelotrichia*, and *Oscillospiraceae*) to indicate contamination. We considered annotations to Clostridia, Clostridiales, *Clostridiaceae*, *Ruminococcaceae*, and *Eubacteriaceae* as consistent with no contamination because at least some *Lachnospiraceae* have previously been annotated as members of these groups in the NCBI database. When calculating (AU)ROC curves in Fig. S2, we scored the first group as 1, the second group as 0, and missing annotations or annotations to “Bacteria” as 0.5.

AUROC statistics were calculated using the pROC package in R (60).

### Flagellar copy number analysis

Copy numbers were obtained using MIDAS2. We first obtained the EggNOG-mapper annotations for all genes in the *L. eligens* pangenome (species #100060 in the MIDAS2 UHGG database). We then filtered these annotations for the characters “flag” in the free text description (case-insensitive). We filtered the copy number matrix to include just these genes, plus the GGDEF-PAS domain protein and diguanylate cyclase that we identified as significant in our PanSweep analysis. Descriptive names for each gene were taken from the EggNOG predicted protein name, except in some cases where there was no name or the name only appeared in the free text description, which were added manually.

To test the associations of gene clusters with cirrhosis, we first clustered the 84 genes using partitioning around medoids (PAM) (61) with Pearson correlation distance. We then selected the number of clusters *k* by maximizing the average silhouette width (ASW) over the range *k* = 2 to *k* = 25, yielding 19 clusters; four clusters had fewer than three genes and were discarded, leaving 15 clusters. For each of these 15 clusters, an eigengene representative was calculated by performing singular value decomposition and taking the first right-singular vector. This eigengene was then tested for an association with cirrhosis using the Wilcoxon rank-sum test; *P*-values were adjusted using the Benjamini-Hochberg method (62) and a false-discovery rate cutoff of 5% was applied.

Copy numbers were visualized using the ComplexHeatmap package (63), using Pearson correlation to hierarchically cluster genes and Euclidean distance to cluster subjects. We also calculated the quantitative enrichment of each gene for cases or controls using the average *t*-statistic from 500 bootstrap samples of the data.

## Data Availability

PanSweep is available as an R package at https://github.com/pbradleylab/pansweep under an MIT license. Code to process data and generate figures is available at https://github.com/pbradleylab/cirrhosis-pansweep and at https://github.com/pbradleylab/Updated_PanSweep_Figure_Code. Processed data for reproducing analyses and the Parquet-formatted UHGP databases can be found at https://zenodo.org/records/14852853.

## SUPPLEMENTAL MATERIAL

Figure S1 msphere.00857-24-s0001.pdf Density plots showing CheckM-estimated percent contamination for genomes containing genes identified as contaminants vs. all other genomes in the same species.

Figure S2 msphere.00857-24-s0002.pdf Receiver-operator characteristic curves showing how well the correlation test, EggNOG-predicted taxonomic ranges, and statistical significance predict contamination, as ascertained via BLAST.

Figure S3 msphere.00857-24-s0003.pdf Heat map showing MIDAS2-estimated copy numbers of flagellar genes in the *L. eligens* pangenome.

Figure S4 msphere.00857-24-s0004.pdf Megablast results for representative non-contaminant, contaminant, and chimeric contaminant genes.

Figure S5 msphere.00857-24-s0005.pdf Discontiguous Megablast results for a representative gene without hits from Megablast.

Supplemental Legends msphere.00857-24-s0006.docx Legends for Table S1 and Fig. S1-S5.

Table S1 msphere.00857-24-s0007.csv An expanded version of Table 1 that also includes the UHGP-90 ID each gene maps to and the total number of genomes it was found in.

ASM does not own the copyrights to Supplemental Material that may be linked to, or accessed through, an article. The authors have granted ASM a non-exclusive, world-wide license to publish the Supplemental Material files. Please contact the corresponding author directly for reuse.
