# Supplementary figures and images for: Small amounts of misassembly can have disproportionate effects on pangenome-based metagenomic analyses
Source: mSphere. 2025 Apr 29;10(5):e00857-24. doi: 10.1128/msphere.00857-24 (PMC12108083; doi:10.1128/msphere.00857-24)

Percent Contamination in Genomes that Contain Significant Genes

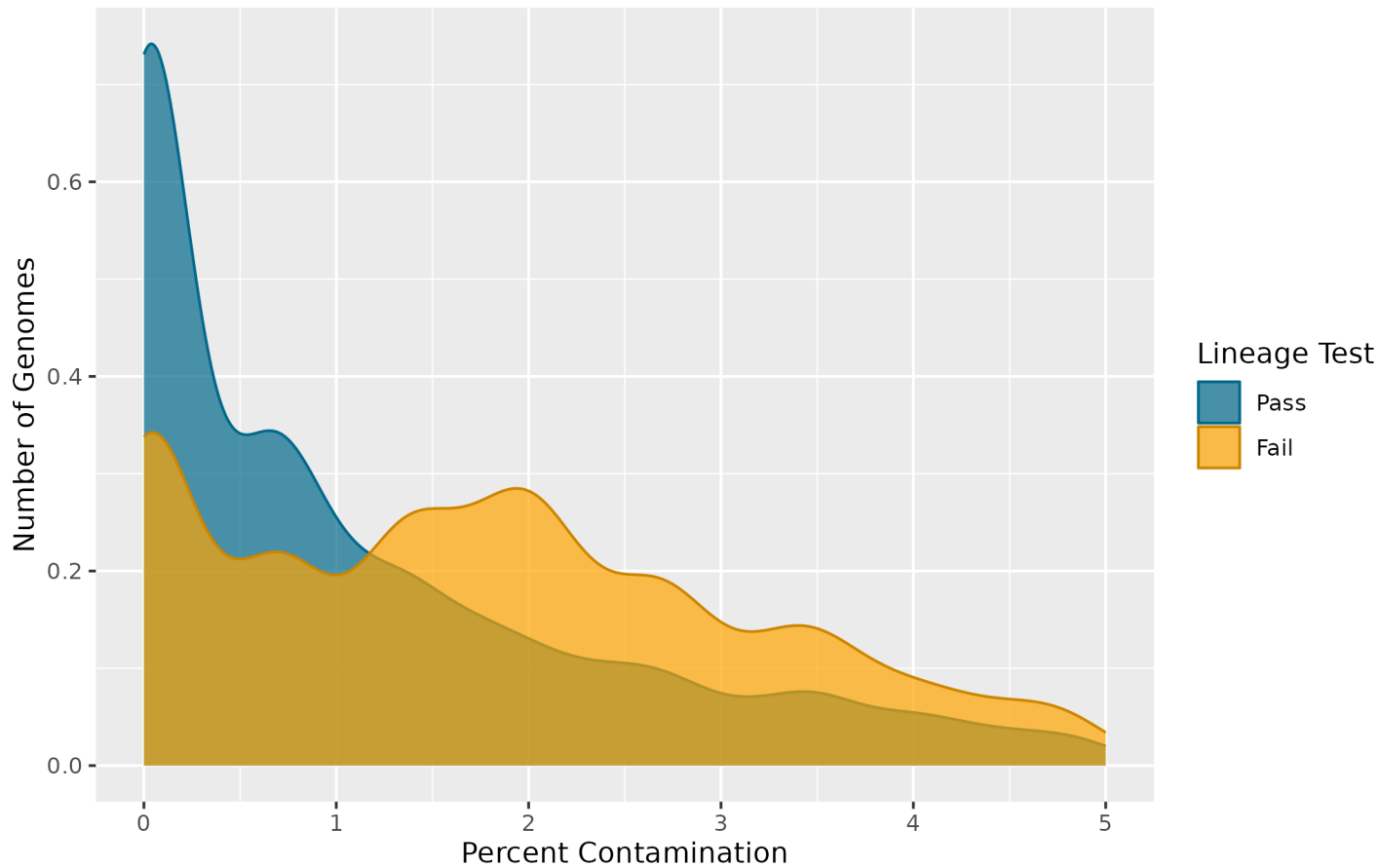

Supplement: Figure S1 — Density plots showing CheckM-estimated percent contamination for genomes containing genes identified as contaminants vs. all other genomes in the same species. [file msphere.00857-24-s0001.pdf]

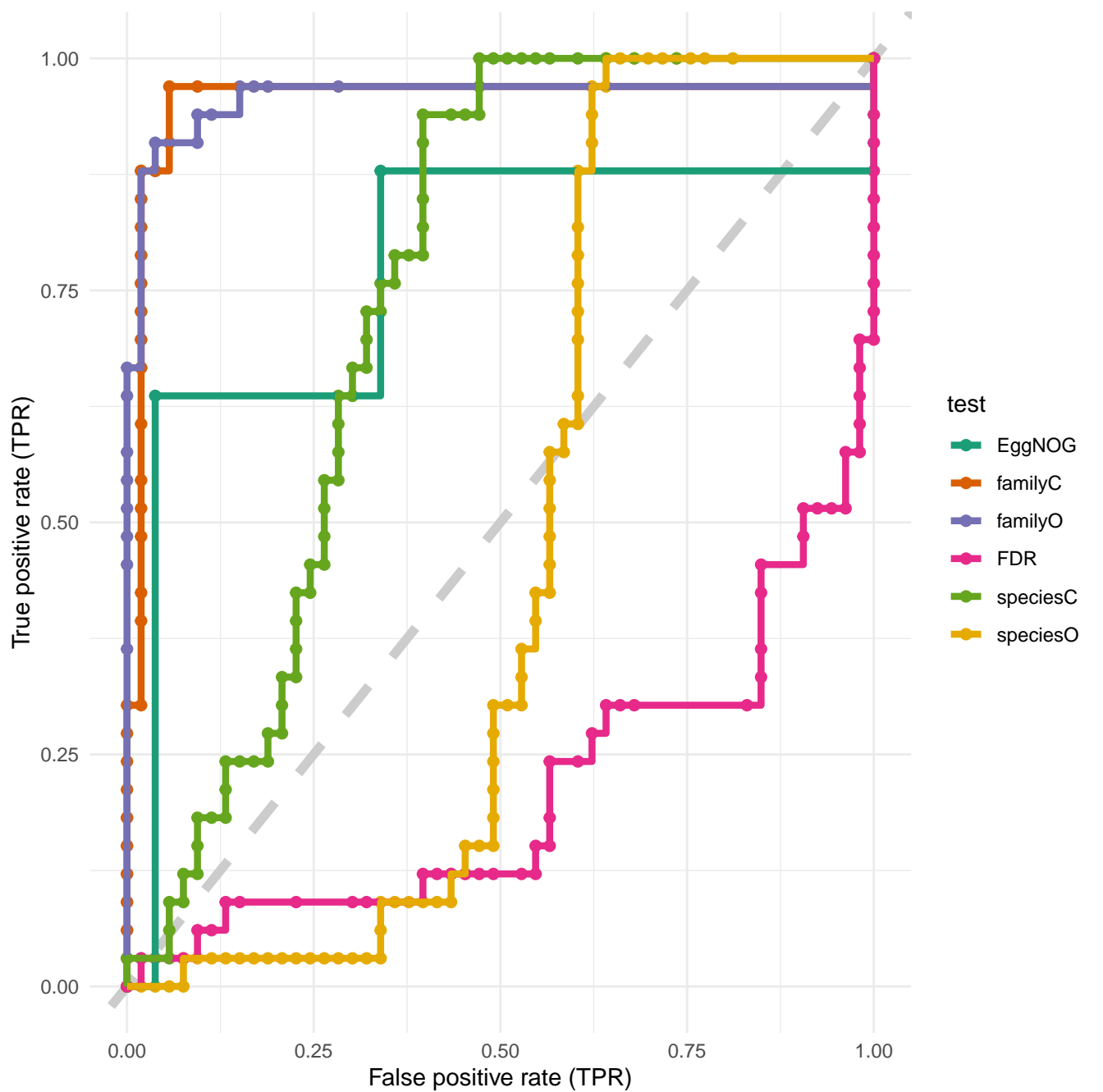

Supplement: Figure S2 — Receiver-operator characteristic curves showing how well the correlation test, EggNOG-predicted taxonomic ranges, and statistical significance predict contamination, as ascertained via BLAST. [file msphere.00857-24-s0002.pdf]

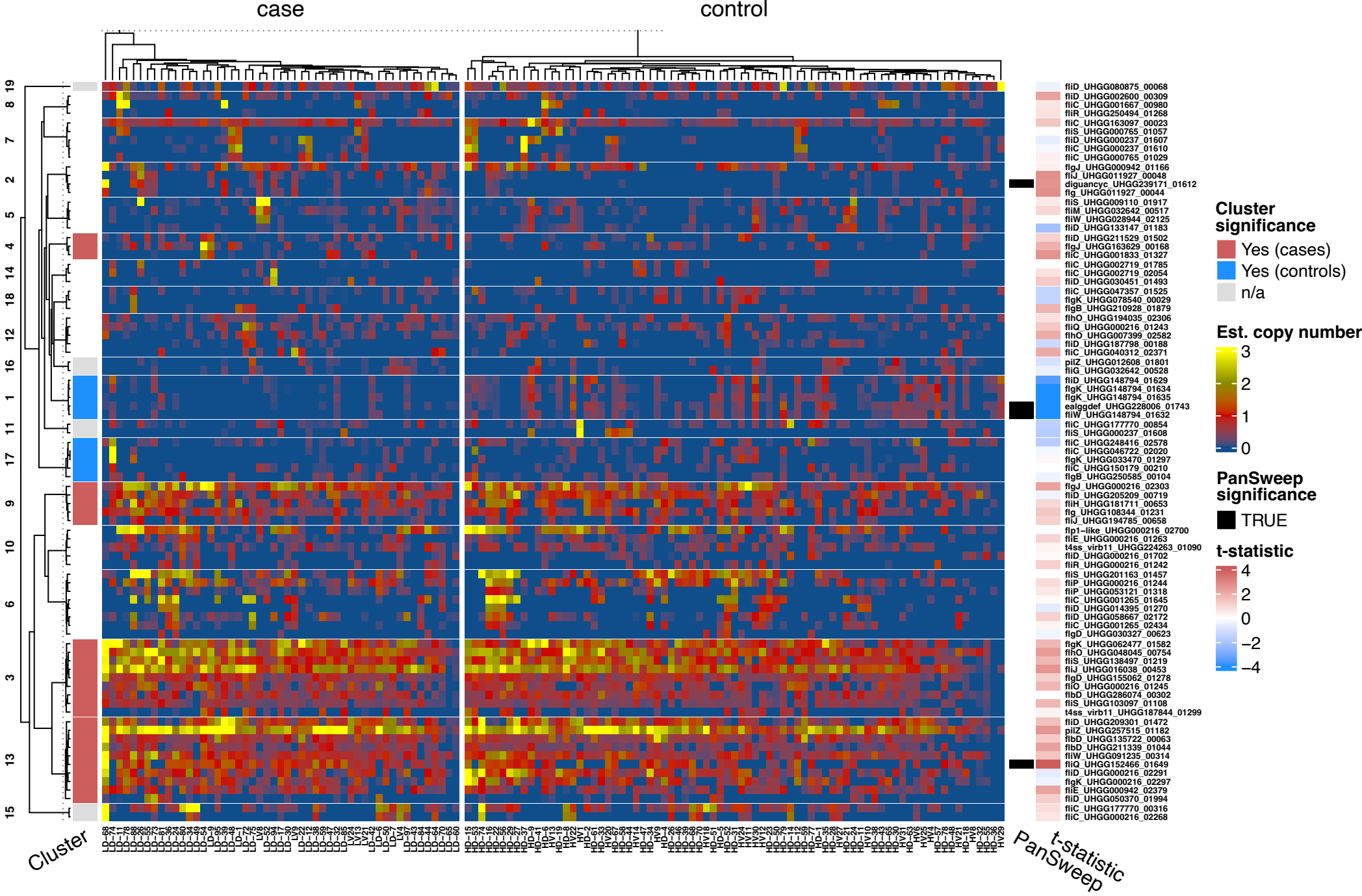

Supplement: Figure S3 — Heat map showing MIDAS2-estimated copy numbers of flagellar genes in the L. eligens pangenome. [file msphere.00857-24-s0003.pdf]

# UHGG000117\_00039: Non-contaminant Gene

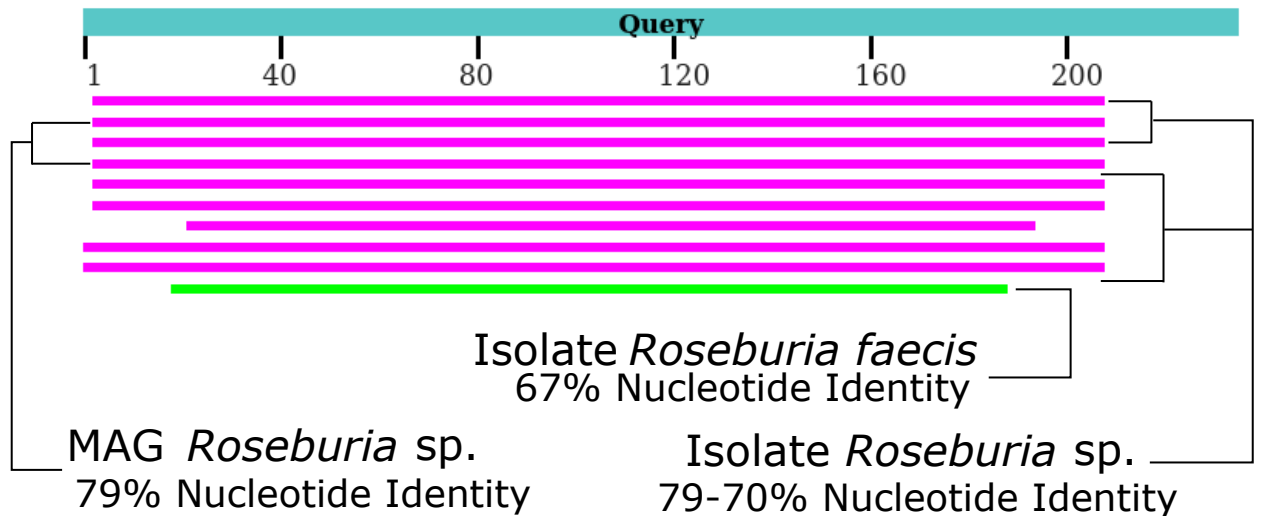

Alignment Scores

■ < 40

■ 40 - 50

■ 50 - 80

■ 80 - 200

■ ≥ 200

Supplement: Figure S5 — Discontiguous Megablast results for a representative gene without hits from Megablast. [file msphere.00857-24-s0005.pdf]
