# Supplementary material for: Small amounts of misassembly can have disproportionate effects on pangenome-based metagenomic analyses
Source: mSphere. 2025 Apr 29;10(5):e00857-24. doi: 10.1128/msphere.00857-24 (PMC12108083; doi:10.1128/msphere.00857-24)
Supplement: Figure S4 — Megablast results for representative non-contaminant, contaminant, and chimeric contaminant genes. [file msphere.00857-24-s0004.pdf]

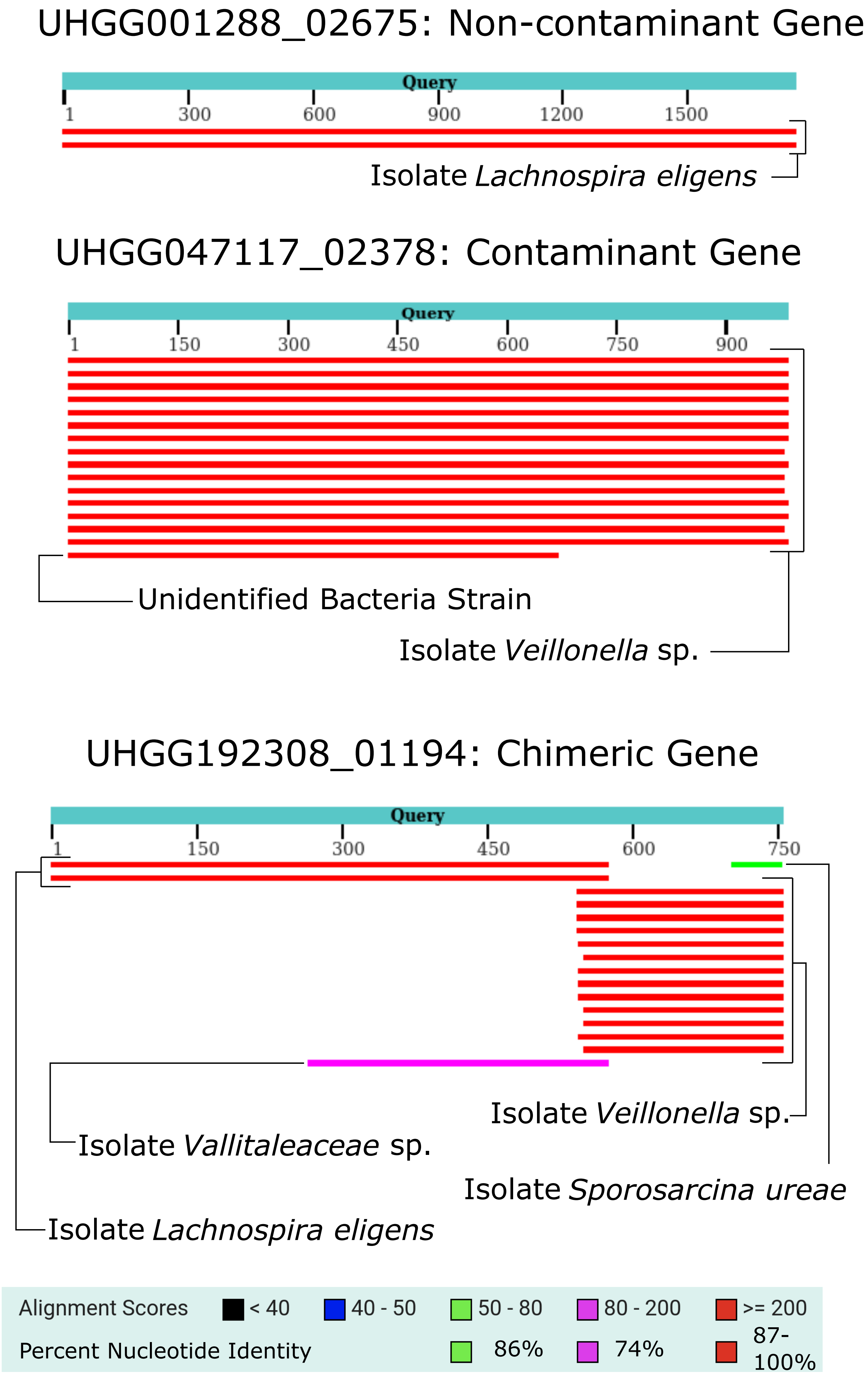
UHGG001288_02675: Non-contaminant Gene
Query
1
300
600
900
1200
1500
Isolate Lachnospira eligens
UHGG047117_02378: Contaminant Gene
Query
1
150
300
450
600
750
900
Unidentified Bacteria Strain
Isolate Veillonella sp.
UHGG192308_01194: Chimeric Gene
Query
1
150
300
450
600
750
Isolate Veillonella sp.
Isolate Vallitaleaceae sp.
Isolate Sporosarcina ureae
Isolate Lachnospira eligens
Alignment Scores
< 40
40 - 50
50 - 80
80 - 200
>= 200
Percent Nucleotide Identity
86%
74%
87-100%
