# Supplementary material for: Small amounts of misassembly can have disproportionate effects on pangenome-based metagenomic analyses
Source: mSphere. 2025 Apr 29;10(5):e00857-24. doi: 10.1128/msphere.00857-24 (PMC12108083; doi:10.1128/msphere.00857-24)
Supplement: Supplemental Legends — Legends for Table S1 and Fig. S1-S5. [file msphere.00857-24-s0006.docx]

# **Supplementary Information**

**Supplementary Table 1:** An expanded version of Table 1 that also includes the UHGP-90 ID each gene maps to, and the total number of genomes it was found in.

**Supplementary Figure 1:** Density plots showing CheckM-estimated percent contamination for genomes containing contaminants vs. all other genomes in the same species.

**Supplementary Figure 2:** Receiver-operator characteristic (ROC) curves showing how well the correlation test (“family” and “species”), EggNOG-predicted taxonomic ranges (“EggNOG”, teal), and statistical significance (“FDR”, purple) predict contamination, as ascertained via BLAST. For the correlation test, we report results for the top-ranked match at the family level or below (blue and red, “family”), as well as the top-ranked match at the species level only (orange and green, “species”). The “overall” version of the correlation test is marked “_o” (blue and orange) whereas the “conditional” version is marked “_c” (red and green).

**Supplementary Figure 3:** Heat map showing MIDAS2-estimated copy numbers of flagellar genes in the *L. eligens* pangenome. Genes are clustered using Pearson correlation and subjects are clustered using Euclidean distance. Genes significantly associated with cirrhosis (Fisher’s test, adjusted p-value ≤ 0.05) are marked in black. The left-side colors show PAM clusters of genes that were significantly associated with cirrhosis (red), controls (blue), or neither (white); clusters with too few genes to perform association tests are colored gray. The right-side “enrichment” colors summarize differences in gene copy number between cases and controls (t-statistic, mean of 500 bootstrap samples; red is enriched in cirrhosis, while blue is enriched in cases).

**Supplementary Figure 4:** Megablast results for representative genes that we manually classified as non-contaminants (UHGG001288_02675, top), contaminants (UHGG047117_02378, middle), and chimeric contaminants (UHGG192308_01194, bottom). Aligned sequences are colored by their alignment score, which we have translated into ranges of percent nucleotide identity.

**Supplementary Figure 5:** Discontiguous Megablast results for a representative gene that had no hits via Megablast but was scored as a non-contaminant (UHGG000117_00039). Aligned sequences are colored by their alignment scores; aligned sequences are directly labeled with their source and with the nucleotide identity range.
